# Hospital Textiles, Are They a Possible Vehicle for Healthcare-Associated Infections?

**DOI:** 10.3390/ijerph9093330

**Published:** 2012-09-14

**Authors:** Sabina Fijan, Sonja Šostar Turk

**Affiliations:** 1 Faculty of Health Sciences, University of Maribor, Žitna ulica 15, 2000 Maribor, Slovenia; 2 Faculty of Mechanical Engineering, Centre for Textile Care, University of Maribor, Smetanova ulica 17, 2000 Maribor, Slovenia; Email: sonja.sostar@um.si

**Keywords:** textile hygiene, disinfection, hospital-acquired infections, inanimate surfaces, infection transmission vehicles

## Abstract

Textiles are a common material in healthcare facilities; therefore it is important that they do not pose as a vehicle for the transfer of pathogens to patients or hospital workers. During the course of use hospital textiles become contaminated and laundering is necessary. Laundering of healthcare textiles is most commonly adequate, but in some instances, due to inappropriate disinfection or subsequent recontamination, the textiles may become a contaminated inanimate surface with the possibility to transfer pathogens. In this review we searched the published literature in order to answer four review questions: (1) Are there any reports on the survival of microorganisms on hospital textiles after laundering? (2) Are there any reports that indicate the presence of microorganisms on hospital textiles during use? (3) Are there any reports that microorganisms on textiles are a possible source infection of patients? (4) Are there any reports that microorganisms on textiles are a possible source infection for healthcare workers?

## 1. Introduction

The occurrence and undesirable complications from healthcare-associated infections have been well recognized in the literature for the last several decades [1]. The most common sources of infectious agents causing healthcare associated infections, described in a scientific review of 1,022 outbreak investigations [2] are (listed in decreasing frequency): the individual patient, medical equipment or devices, the hospital environment, the healthcare personnel, contaminated drugs, contaminated food, and contaminated patient care equipment. Although the person-to-person transmission route is the most likely, the role of the environment should not be ignored and hospital linen may contribute to the spread of nosocomial infections [3,4]. 

Healthcare textiles include bed sheets, blankets, towels, personal clothing, patient apparel, uniforms, gowns, drapes for surgical procedures [5]. Contaminated textiles and fabrics often contain high numbers of microorganisms from body substances, including blood, skin, stool, urine, vomitus, and other body tissues and fluids. Although contaminated textiles in healthcare facilities can be a source of substantial numbers of pathogenic microorganisms, reports of healthcare associated diseases linked to contaminated fabrics are few, therefore the overall risk of disease transmission is very low [5]. 

Cleaning in general has two main functions: first: non-microbiological, to improve or restore appearance, and prevent deterioration. Second, microbiological, to reduce the numbers of microbes present, together with any substances that support their growth or interfere with disinfection [4]. The purpose of laundering hospital textiles is therefore to ensure clean and safe textiles for patients and staff and thus enable uninterrupted implementation of healthcare [5,6]. The most common found microorganisms on hospital textiles are: Gram negative bacteria, coagulase negative staphylococci, *Bacillus* sp. and typical skin flora [7].

Most people working in hospitals assume that laundry returned to them is in fact clean and therefore safe. Laundry may certainly have had the dirt removed, but it is far from sterile and experience encourages infection control teams to take laundering very seriously in outbreaks that seem to have no obvious cause [8].

## 2. Reports on the Survival of Microorganisms on Hospital Textiles after Laundering

Literature in the field of survival of microorganisms on hospital textiles after laundering is very diverse and perhaps even confusing and contradictory. Each publication states a different laundering temperature as appropriate. It is therefore important to note that a successful laundering procedure is dependent on several factors and each much be optimized. These factors with a possible synergistic effect include: duration of laundering procedure, mechanical action of laundering procedure, dosage and type of added detergents and disinfection agents, bath ratio, type of linen, filling ratio, *etc*. According to Sinner the four basic interconnected factors of the laundering procedure are: duration, mechanical action, chemicals and temperature [9]. If one of these factors is decreased such as for example temperature, then other factors such as chemicals, mechanical action or time must be increased to achieve the same laundering and disinfecting effect. This also explains the differences in the published efficient laundering conditions. The exact correct optimized combination of all the mentioned factors is therefore important in order to achieve a hygienic laundering procedure for hospital textiles. Wilcox and Jones [10] stated that many isolates of *Enterococcus faecium* survived exposure to laundering temperatures specified in the U.K. Department of Health guidelines for disinfecting foul and used and infected linen (60 °C for 10 min). Another published report by Orr and co-workers [11] even confirms survival of certain strains of enterococci at laundering temperatures as high as 71 °C. They therefore concluded that hospital linen is a possible source of enterococcal cross-infection. 

The survival of enterococci on textiles at laundering temperatures as high as 60 °C was also confirmed by a study [12] where biomonitors (*Enterococcus faecium* inoculated onto textile swatches with pre-inoculated defibrinated sheep blood) were washed in a simulated common hospital laundry procedure. It was found that *Enterococcus faecium*, as well as *Staphylococcus aureus*, *Enterobacter aerogenes* and *Pseudomonas aeruginosa* all survived the chosen laundering conditions at 60 °C, but none of the challenge organisms survived laundering at 75 °C. On the other hand other studies [13,14] confirm that optimizing the laundering procedure including using high-tech environmental detergents and innovative disinfection agents renders an appropriate disinfection effect, even at laundering temperatures as low as 30 °C as noted in the study [13] with challenge organisms *Enterococcus faecium* and *Enterobacter aerogenes.* In another study [14] the optimum laundering temperature was found to be at 40 °C. 

Walter and co-workers [15] reported that *Staphylococcus aureus* survived a 10 min laundering at 54 °C followed by drying. *Klebsiella pneumoniae* also survived the same laundering procedure, but did not survive the drying procedure. The same research also indicated that the challenge bacteria *Staphylococcus aureus* was not found after a 60 °C laundering procedure; thus recommending a 60 °C laundering procedure for linen in healthcare facilities. 

In the report by Smith and co-workers [16] it was found that soiled hospital terry towels initially contaminated with Gram-positive rods (predominantly *Klebsiella*, *Enterobacter* and *Serratia* spp.) and Gram-positive bacteria (predominantly *Staphylococci*) in the range between 10^7^ to 10^9^/100 cm^2^ were washed in different laundering procedures. It was found that the washing cycle with a temperature of 60 °C followed by the drying cycle at 93.3 °C was sufficient to maintain linen hygiene. 

Christian and co-workers [17] also conducted experimental research on low temperature laundering of hospital textiles using economically reasonable chemicals and wash conditions. They examined the disinfection effect of laundering procedures against aerobic chemoorganotrophs, staphylococci and total coliforms. They found that low temperature washing procedures (47.8 °C) using increased concentrations of bleach eliminated all bacterial groups as effectively as the high temperature procedures (77 °C). 

In the research by Blaser and co-workers [7] a comparison of laundering procedures at 71.1 °C and 22 °C was conducted. The argument of such low-temperature washing was the vast amount of energy used for laundering at 71.1 °C. The 22 °C laundering procedure included the use of low-temperature chemicals. The initial counts on the use soiled terry towels and sheet were between 10^6^ to 10^8^/100 cm^2^ with predominantly Gram-negative rods (especially *Enterobacteriaceae* and *Pseudomonadaceae*) and *Staphylococcus* species as the most common Gram-positive organisms. It was found that the bacterial counts from low-temperature and high-temperature washed fabrics were comparable. The authors therefore concluded that low-temperature washing for eliminating pathogenic bacteria from hospital laundry is as effective as high-temperature laundering. 

It has been reported that *Clostridium difficile* [18] spores can survive temperatures and chemical treatment of typical hospital laundering cycles and that cross-contamination of *Clostridium difficile* spores can occur on bed linen during a wash cycle. Therefore the persistent nature of this organism must be considered by infection control personnel when implementing programs for laundering soiled and contaminated hospital linen. Articles reporting on the survival of microorganisms on hospital textiles after laundering, together with their main conclusions are summarized in Table 1. 

**Table 1:** Reports on the survival of microorganisms on hospital textiles after laundering.

Described laundering conditions	Added disinfection agent or bleach	Surviving microorganism	Reference
10 min at 60 °C	No	*Enterococcus faecium*	Wilcox & Jones, 1995 [10]
10 min at 60 °C or 3 min at 71 °C	No	Certain strains of *Enterococcus faecalis* and *Enterococcus faecium*	Orr *et al*. 2002 [11]
less than 10 min at 60 °C	3 mL Peroxyacetic acid/ kg textiles	*Enterococcus faecium, Staphylococcus aureus, Pseudomonas aeruginosa* and *Enterobacter aerogenes*	Fijan *et al*. 2007 [12]
20 min at 30 °C	10 mL Sodium hypochlorate/kg textiles or 12.5 mL peroxyacetic acid/kg textiles	*Enterococcus faecium* and *Enterobacter aerogenes*	Fijan *et al*. 2010 [13]
43 min at 30 °C	10 mL Sodium hypochlorate/kg textiles	*Enterococcus faecium*	
13 min at 49 °C	Added chlorine bleach (without specifications)	*Staphylococcus aureus* and *Klebsiella pneumoniae*	Walter *et al*. 1975 [15]
66 °C	Added chlorine bleach cycle (without specifications)	*Staphylococci*, *Klebsiella,* and *Enterobacter* species	Smith *et al*. 1987 [16]
8 min at 47.8 °C	0.58 Chlorine bleach/kg	Predominantly aerobic bacteria, staphylococci and total coliforms	Christian *et al*. 1983 [17]
77.2 °C	0.11 Chlorine bleach/kg		
22.2 °C	Low temperature bleach (without specifications)	Predominantly *Enterobacteriaceae*, *Pseudomonadaceae* and *Staphylococcus* species	Blaser *et al*. 1984 [7]
71.1 °C	High temperature bleach (without specifications)		
Typical program for hospital bed linen	50 ppm Chlorine, 54 ppm peracid, 100 ppm peroxid	*Clostridium difficile* spores	Hellickson & Owens, 2007 [18]

## 3. Reports on the Presence of Microorganisms on Hospital Textiles

Table 2 summarizes reports of articles on the presence of microorganisms on hospital textiles.

**Table 2:** Reports on the presence of microorganisms on hospital textiles.

Surviving microorganism	Hospital textile	Time	Reference
Moulds	Sheets, pyjamas	After use by patients	Bureau-Chalot *et al*. 2004 [3]
Coagulase-negative staphylococci, *Bacillus* spp., *Corynebacterium* spp., saprophytic Gram negative bacilli	Sheets, pyjamas, uniforms	After laundering in hospital laundry	Fijan *et al*. 2005 [6]
*Staphylococcus aureus, Clostridium difficile* and vancomycin resistant enterococci	Nurses’ uniforms	After 24 h shift	Perry *et al*. 2001 [19]
*Acinetobacter baumannii*	Bed linen and curtains	After use	Hota *et al*. 2004 [20]
MRSA	Bed linen and uniforms		
Coagulase negative Staphylococci, *Corynebacterium* spp., *Micrococcus* spp., *Bacillus* spp., *Enterococcus* spp., saprophytic Gram negative bacilli, moulds	Sheets, pyjamas and uniforms	After laundering in hospital laundries	Fijan *et al*. 2005 [21]
Rotaviral RNA	Sheets, pyjamas and uniforms	After laundering in hospital laundries	Fijan *et al*. 2008 [22]
Parainfluenza virus	Hospital gown	4 h after inoculation	Brady *et al*. 1990 [23]
Vancomycin resistant enterococci	Bed linen	11 weeks after inoculation	Hochmuth *et al*. 2005 [24]

The report by Brady [23] indicates that the parainfluenza virus can survive 4 h on clothing; thus suggesting the need to consider fomites as a possible source of transmission of the virus. In the report by Perry and co-workers [19] microbiological sampling of nurses’ uniforms yielded the detection of *Staphylococcus aureus*, *Clostridium difficile* and vancomycin-resistant enterococci (VRE) before and after the span of duty. The authors recommended provision and frequent changing of nurses’ uniforms.

The report by Hochmuth and co-workers [24] noted that VRE strains can survive for 11 weeks on linen and plastic with a 4 log cfu reduction after 7 weeks. They concluded that VRE can survive for prolonged periods on inanimate surfaces that are frequently encountered in a healthcare setting and that the proper disinfection of these surfaces is important in the prevention of nosocomial transmission of VRE.

In the report by Bureau-Chalot and co-workers [3] over 200 samples of hospital linen (sheets, pyjamas) as well as linen rooms and trolleys for transporting linen were collected. The most common found microorganisms were of human origin (coagulase-negative staphylococci) and of environmental origin (*Bacillus* spp., moulds). It was found that clean linen can become a vector for transmission of pathogens or that pathogens present on linen may become airborne during bed-making and may then contaminate surfaces.

The report by Hota [20] also reviews the presence of microorganisms on hospital textiles. In her survey of the literature *Acinetobacter baumannii* was found on bed linen and curtains, as well as other parts of the surrounding inanimate environment [25], MRSA was found on uniforms worn by health workers and on bed linen [26,27]. Other environmental sites that included VRE were gowns worn by patients and health workers [28].

There are several published articles which show results of investigating the microbial counts of laundered hospital linen using contact plates with RODAC agar and swabbing over period of 5 years between 2004 and 2008 [6,21,22]. The following microorganisms at various occasions were found in hospital laundries on cleaned, folded laundry prepared for reuse: coagulase negative *Staphylococci*, *Corynebacterium* spp., *Micrococcus* spp., *Bacillus* spp., non-fermentative Gram negative bacilli, *Enterococcus* spp., saprophytic Gram negative bacilli, moulds and rotaviral RNA. Although the results seem alarming, after the initial microbiological-sanitary surveillance, all laundries underwent systematic sanitary measures and the results of microbial investigations yielded very low counts on the clean and folded hospital textiles.

## 4. Reports of Microorganisms from Hospital Textiles as a Possible Source of Infection of Patients

Reports on hospital textiles as possible source of infection of patients are summarized in Table 3.

**Table 3:** Reports on hospital textiles as possible source of infection of patients.

Microorganism	Hospital textile	Reference
*Streptococcus pyogenes*	Babies’ vests (contamination of dryers)	Brunton, 1995 [8]
*Bacillus cereus*	Cleaned hospital linen	Barrie *et al*. 1994 [29]
	Cleaned hospital linen	Barrie *et al*. 1992 [30]
	Cleaned infants’ nappies	Birch *et al*. 1981 [31]
	Reused towels	Dohmae *et al*. 2008 [32]
	Towels and bedsheets	Sasahara *et al*. 2011 [33]
MRSA	Bed linen	Creamer & Humphreys, 2008 [34]
	Linen	Shiomori *et al*. 2002 [35]
*Pseudomonas aeruginosa*	Patients’ clothes, bed linen	Panagea *et al*. 2005 [36]
VRE	Drawsheet	Bonten *et al*. 1996 [37]
*Staphylococcus aureus*	Mattress	Ndawula & Brown, 1991 [38]
Antibiotic resistant coliform bacilli	Blankets, mattresses	Kirby *et al*. 1956 [39]
*Trichophyton interdigitale*	Contaminated socks	English *et al*. 1967 [40]

An extensive investigation [8] of what seemed to be a recurring outbreak of streptococcal infection associated with a maternity unit was conducted. On each occasion, extensive environmental and epidemiological investigations were carried out, which indicated that babies were being infected very shortly after birth. The infection team decided to look at the laundering of the vests usually given to new-born children. Investigation of the laundry and in particular the hot air dryers, revealed extensive contamination with the MT type of *Streptococcus pyogenes* involved in the outbreak. After all babies’ vests had been autoclaved the outbreaks ceased.

An investigation into two cases of post-operative *Bacillus cereus* meningitis [29,30] revealed that hospital linen laundered by a batch continuous washing machine was heavily contaminated by *Bacillus cereus* spores. It was found that the linen introduced into the washing machine had a high *Bacillus cereus* spore content and that this was still present after the wash process. In a maternity unit 44% of umbilical swabs from neonates contained an unusual serotype of *Bacillus cereus* [31]. On further investigation the same serotype could be isolated from air samples, the hands of members of staff and ‘clean’ nappies from the hospital laundry. It was found that the nappies appeared to be the primary vehicle of *Bacillus cereus* dissemination among the infants.

Creamer and Humphreys [34] emphasized that bed linen can rapidly become heavily contaminated with colonised skin scales and may contribute to the spread of infections. They also stated that precautions such as changing of linen after discharge, using national or standard laundering procedures, storage of clean linen in clean linen storage presses, clean trolleys, *etc*. are sufficient. In their literature survey they found MRSA [35], *Pseudomonas aeruginosa* [36], VRE [37] to have been associated with the spread of pathogens by bed linen as one of the possible environmental routes.

Ndawula and Brown [38] found mattresses were reservoirs of epidemic methicillin-resistant *Staphylococcus aureus*. In a study [39] of the cause of urinary tract infections the authors were unable to determine the source of the resistant bacteria and the exact mode of infection; the catheters themselves, and the solutions used to irrigate them, could not be incriminated. Blankets, mattresses, and possibly the nasopharyngeal flora of hospital personnel appeared more likely possibilities.

Several *Bacillus cereus* nosocomial infections in Japan were investigated [32] and novel multilocus sequence types were found in patients. After eliminating food-poisoning as a causative agent it was found that the similar strains were found on dried and streamed reused towels and that towels represent an important source of contamination.

In the investigation of a *Bacillus cereus* bacteremia outbreak [33] it was found that hospital linens and the washing machine were highly contaminated with *B. cereus*, which was also isolated from the intravenous fluid of symptomatic patients. All of the contaminated linens were autoclaved, the washing machine was cleaned with a detergent, and improved hand hygiene was promoted among the hospital staff. The number of patients per month that developed new *B. cereus* bacteremia rapidly decreased after implementing these measures. The source of this outbreak was identified as *B. cereus* contamination of hospital linens, and *B. cereus* was being transmitted from the linens to patients via catheter infection. The authors concluded that their findings demonstrated that bacterial contamination of hospital linens can cause nosocomial bacteremia.

In a study of a significantly higher incidence of *Trichophyton rubrum* along with a common incidence of *Trichophyton interdigitale* in a long-stay hospital for mentally retarded men it was found that a significant number of crippled patients who had never walked acquired tinea pedis [40]. The appropriate fungus was isolated before laundering from the worn socks of three patients with *T. interdigitale* infection and of one patient with *T. rubrum* infection. After laundering the fungus was recovered from the socks of one of the patients with *T. interdigitale* infection. In view of the failure of laundering to eliminate the fungus from worn socks, it was suggested that infected socks were the most important route of cross-infection among the crippled patients.

All these research publications emphasise that correct laundering procedures of hospital textiles are an important measure for preventing health-acquired infections especially when other more common sources of infections have been ruled out [8].

## 5. Reports on Microorganisms on Textiles as a Cause for Nosocomial Infections of Hospital Workers

Reports on hospital textiles as possible source of infection of hospital workers are summarized in Table 4.

**Table 4:** Reports on hospital textiles as possible source of infection of hospital workers.

Microorganism	Source	Employee	Reference
*Sarcoptes scabiei*	Handling unclean hospital linen	Hospital laundry personnel	Thomas *et al*. 1987 [41]
*Microsporum canis*	Handling contaminated laundry	Hospital staff	Shah *et al*. 1988 [42]
*Salmonella typhimurium*	Handling unclean hospital sheets	Hospital laundry personnel	Datta & Pridie, 1960 [43]
*Salmonella hadar*	Handling unclean hospital linen	Hospital laundry personnel	Standaert *et al*. 1994 [44]
Hepatitis A virus	Handling unclean hospital linen	Hospital laundry personnel and nurses’ aids	Borg & Portelli, 1999 [45]
			Keeffe, 2004 [46]

Wilson and co-workers [47] did not find any evidence to support the hypothesis that uniforms could be a vehicle for the transmission of infections as no studies demonstrated the transfer of microorganisms from uniforms to patients in a clinical situation. They state however that there is an epidemiological link between contaminated clothing and healthcare associated infection when clothing is highly contaminated in an industrial laundry.

In an outbreak of scabies among the employees of a hospital laundry [41] it was found that the most probable cause of this outbreak was transmission via unclean hospital bed linen. During the time compatible with the outbreak a patient with Norwegian scabies was hospitalized. It was concluded that improper handling of the dirty laundry by laundry workers (alleged lack of use of protective gloves) led to the outbreak among the laundry workers.

Shah and co-workers [42] reported of an unusual nosocomial outbreak among staff and patient infected with *Microsporum canis*. It was established that likely modes of subsequent disease transmission from a single infected patient included person-to-person contact and handling of contaminated laundry.

In an outbreak of infection with *Salmonella typhimurium* [43] in a general hospital extensive research was conducted to find the source of transmission. There was no evidence of food poisoning and it was found that several laundry workers who handled sheets from infected persons were excreting *S. typhimurium*. These laundry workers did not have any direct contact with infected patients. Therefore handling dirty laundry was the most likely cause of infection among these laundry workers. The same conclusion was reached by Standaert and co-workers [44], who investigated an extensive outbreak of salmonella gastroenteritis in a nursing home among residents as well as employees (nurses and laundry personnel). Three laundry personnel who had no contact with residents were infected. Due to the delayed onset of symptoms of these laundry personnel; a secondary transmission was suggested. It was concluded that linen soiled with faeces was the source of nosocomial *Salmonella hadar* infection among the laundry workers. It was found that most of these laundry personnel did not use protective clothing and gloves when handling dirty and infected laundry. The authors stressed the importance of using appropriate precautions when handling dirty linen.

Borg and Portelli [45] investigated laundry personnel and nursing aids in pediatric and infectious disease wards for seropositivity to hepatitis A. It was found that the ratio for seropositivity to hepatitis A between laundry personnel consistently handling dirty linen as compared with colleagues handling only clean items was 16.5. The authors concluded that the increased exposure of hospital laundry workers to potentially infected linen can constitute a risk of occupational hepatitis A for this group of employees. Keeffe [46] also listed laundry workers as one of the at-risk occupations for a hepatitis A infection.

Oliphant and co-workers [48] also investigated an outbreak of Q fever among laundry workers handling material from a laboratory and found that it was presumably transmitted from contaminated clothing.

From all these publications it is obvious that it is necessary to implement infection control practices including proper handling of dirty linen by laundry workers in order to prevent possible health-acquired infections [49,50]. Although soiled linen may contain large numbers of pathogenic microorganisms, the risk of actual disease transmission is very low and hygienic and common-sense storage and processing of clean and soiled linen are recommended [51].

## 6. Discussion and Conclusions

Healthcare institutes are obligated to ensure all necessary measures to prevent or limit the spread of healthcare associated infections. One of the possible vehicles of transmission is inanimate fomites such as textiles.

When textiles are heavily contaminated with potentially infective body substances, they can contain bacterial loads of 10^6^ to 10^8^ cfu/100 cm^2^ of fabric. However, the incidence of healthcare associated infections transmitted from hospital linen is very low especially when evaluated in the context of the volume of items laundered in healthcare settings (estimated to be five billion pounds annually in the United States) [5]. It is obvious that the various existing control measures for hospital laundry are effective in reducing the risk of disease transmission to patients and staff. Therefore, use of current control measures should be continued to minimize the contribution of contaminated laundry to the incidence of healthcare associated infections. These control measures are based on principles of hygiene, common sense, and consensus guidance.

According to the Slovenian Public Gazette [52] the correct hygienic management of hospital textiles is achieved by minimal technical requirements regarding sorting, transport and laundering textiles. These requirements according to the Slovenian Expert background and guidelines for management and prevention of health-associated infections include [53] the following measures:

1. Correct collecting and sorting of contaminated hospital textiles;
2. Correct transporting of contaminated hospital textiles;
3. Correct division of clean and unclean area in laundry;
4. Correct sorting, laundering, drying and ironing of hospital textiles;
5. Correct transport and storage of clean hospital textiles.

Very similar conditions are defined in the U.S. Recommendations of C.D.C. and Healthcare Infection control practices Advisory Committee [5], the USA APIC text of infection control and epidemiology [54] as well as the standard EN 14065 RABC: Risk Analysis and Biocontamination Control System for textiles in Europe [55]. These conditions are also implemented in the German Quality and Hygiene assurance for hospital textiles RAL-GZ 992 [56]. The RABC standard and the RAL-GZ 992/2 quality and hygiene assurance are valid as a system of quality hygiene assurance for hospital textiles in Slovenia. Experience has been shown that proper implementing of any of these chosen Guidelines results in clean and properly disinfected linen in healthcare facilities. The key elements in the laundering process according to the APIC text [54], supported by the U.S. C.D.C. [5], include water temperature, type of detergents, disinfectant, rinsing and finishing as well as supplementing the process with common sense and hygienic approaches to collection and transport.

According to the C.D.C. guidelines [5] it is also important to acknowledge that hospital textiles (especially high tough surfaces such as bed linen and pajamas) should not only be appropriately cleaned, but also disinfected and in certain cases (such as surgical drapes and reusable gowns, and in some cases linens in neonatal intensive care units as well as linens in burn therapy units) even sterilization of textiles is necessary [5].

In the future more research should be conducted in the area of the adherence of microorganisms onto textiles and the likelihood of shedding from the textiles during use thus making them airborne. Another important theme for future research is to study the infectivity of microorganisms after being adhered onto textiles for certain periods of time. This information would give more insight in the transfer of microorganisms from textiles to patients in a clinical situation. Another important future focus is using particles with antimicrobial activity for textile modification in order to enhance antimicrobial properties of medical textiles without adding antimicrobial agents into textiles which can have possible harmful or toxic effects [57].
